# Motif-directed network component analysis for regulatory network inference

**DOI:** 10.1186/1471-2105-9-S1-S21

**Published:** 2008-02-13

**Authors:** Chen Wang, Jianhua Xuan, Li Chen, Po Zhao, Yue Wang, Robert Clarke, Eric Hoffman

**Affiliations:** 1 Department of Electrical and Computer Engineering, Virginia Polytechnic Institute and State University, Arlington, VA, USA; 2 Research Center for Genetic Medicine, Children's National Medical Center, Washington, DC, USA; 3 Departments of Oncology and Physiology & Biophysics, Georgetown University School of Medicine, Washington, DC, USA

## Abstract

**Background:**

Network Component Analysis (NCA) has shown its effectiveness in discovering regulators and inferring transcription factor activities (TFAs) when both microarray data and ChIP-on-chip data are available. However, a NCA scheme is not applicable to many biological studies due to limited topology information available, such as lack of ChIP-on-chip data. We propose a new approach, motif-directed NCA (mNCA), to integrate motif information and gene expression data to infer regulatory networks.

**Results:**

We develop motif-directed NCA (mNCA) to incorporate motif information into NCA for regulatory network inference. While motif information is readily available from knowledge databases, it is a "noisy" source of network topology information consisting of many false positives. To overcome this problem, we develop a stability analysis procedure embedded in mNCA to resolve the inconsistency between motif information and gene expression data, and to enable the identification of stable TFAs. The mNCA approach has been applied to a time course microarray data set of muscle regeneration. The experimental results show that the inferred TFAs are not only numerically stable but also biologically relevant to muscle differentiation process. In particular, several inferred TFAs like those of MyoD, myogenin and YY1 are well supported by biological experiments.

**Conclusion:**

A novel computational approach, mNCA, has been developed to integrate motif information and gene expression data for regulatory network reconstruction. Specifically, motif analysis is used to obtain initial network topology, and stability analysis is developed and applied with mNCA to extract stable TFAs. Experimental results on muscle regeneration microarray data have demonstrated that mNCA is a practical and reliable computational method for regulatory network inference and pathway discovery.

## Background

High-throughput biological data provide a powerful opportunity to study genome systems from a global perspective that may lead to a better understanding of their underlying biological processes [1]. In recent years, many computational methods have been proposed to identify gene modules, interactions and pathways in biological systems [2-5]. Most methods assume that the expression activity of an entire gene population results from a much smaller number of latent factors such as transcription factors. This assumption not only coincides with the modular view of biological systems, but it also makes the computational task much easier [2]. For gene regulatory network modeling, there are two major trends in the literature: the first trend is to use clustering methods to explore the similarity in expression patterns [2], whereas the second trend uses decomposition methods to infer latent (hidden) factor activities [3-5].

The results of pure computational approaches are often difficult to interpret due to the lack of biological knowledge support. Biological regulatory systems are complex in nature, and key activities may occur simultaneously in the genome, transcriptome and proteome. Hence, any computational model based only on mRNA measurements may be too simple to describe the entire system. Recently, many researchers have tried to integrate multiple data sources to infer and reconstruct biological networks. For example, network component analysis (NCA) is a topological knowledge based algorithm that utilizes both protein binding data and gene expression data to reveal underlying transcription factor activities [6]. NCA has been shown to be effective in finding cell cycle regulators in yeast [7]. Despite its success in yeast data, some issues prevent NCA to infer regulatory networks other than in yeast. First, complete biological connection data, such as high-throughput ChIP-on-chip data, are often not available for common species including rodent and human. Second, when different heterogeneous data sources are integrated for computational inference, the consistency of different data sources is often not guaranteed. Third, since topological knowledge (network connections) also comes from biological experiments, this knowledge likely also contains many false-positives/negatives that can lead to incorrect network inference.

In this paper, we propose a motif-direct NCA (mNCA) approach for regulatory network inference. First, for species with no high-throughput ChIP-on-chip data, possible network connections can be constructed by finding transcription factors and their potential binding sites in genes' promoter regions. Our rationale is that TF-gene regulation occurs only after TFs bind to specific regulatory elements (DNA sequence motifs) in a gene's promoter region. Second, with the awareness of false-positives/negatives contained in motif information, a stability analysis procedure will be developed for mNCA, not only to test the consistency between motif information and microarray data, but also to evaluate the reliability of the estimated transcription factor activities (TFAs). The new scheme has been applied to a muscle regeneration microarray data set for regulatory network inference. With the stability analysis of mNCA, several reliable TFs have been identified as key regulators of muscle differentiation.

## Methods

### Network component analysis (NCA)

**Figure 1:**
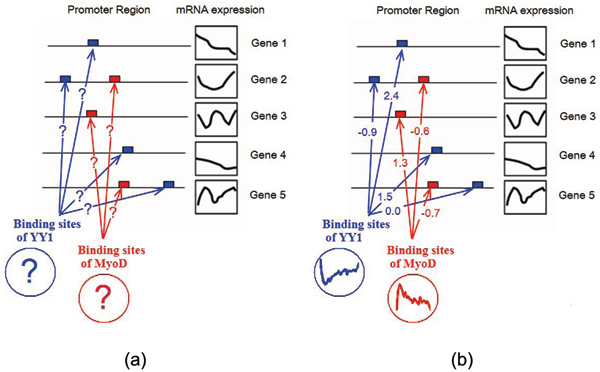
An illustrative example for the NCA approach as in muscle regeneration studies. The network topology is formed by the connection matrices of the transcription factors (TFs) such as YY1 and MyoD to their target genes as shown in (a). The main objective of the NCA approach is to estimate the transcription factors' activities (TFAs) and their target genes via the estimated connection matrices as shown in (b).

Network Component Analysis (NCA) is a computational method to infer latent factors and the connection relationship of a network, given the initial topology (connection) information and the measurement of gene expression. In Fig. 1, we illustrate the NCA approach with an example from muscle regeneration studies [8]. The mathematical model of NCA can be formulated as

$$\begin{array}{c} E_{N\times M}=A_{N\times L}T_{L\times M},\\ s.t.\;A\in Z_{0}, \end{array}$$

where **E **is the observation, *A *connection matrix, **T **latent factors, and *Z*_0 _the initial topology of the network. L is the number of latent (hidden) factors, M the number of experiment conditions, and N the number of genes. As illustrated in Fig. 1, the latent factors are the transcription factors such as YY1 and MyoD; the network topology is formed by the connection matrices of the TFs to their target genes. The main objective of the NCA approach is to estimate the transcription factors' activities (TFAs) and their target genes. The NCA optimisation criterion can be simply denoted as [6]:

$$\begin{array}{c} min||E_{N\times M}-A_{N\times L}T_{L\times M}|{|}^{2},\\ s.t.\;A\in Z_{0}. \end{array}$$

The NCA algorithm was originally developed for gene regulatory network reconstruction. The model (1) can be interpreted in this way: the *N *genes' expression pattern under *M *different conditions can be seen as a combination effect of *L *transcription factors (TFs). Note that it is well accepted that a linear model only holds after log-ratio transform [6]:

log(**E***r*_*N*×*M*_) = *A*_*N*×*L*_log(**T***r*_*L*×*M*_),

where **E***r*_*ij *_= *E*_*ij*_(*t*)/*E*_*ij*_(0) (*i *= 1,...,*M*; *j *= 1,...,*N*) and **T***r*_*kl *_= *T*_*kl*_(*t*)/*T*_*kl*_(0) (*k *= 1,...,*L*; *l *= 1,...,*M*) are ratios of gene expression values and transcription factor activities (TFAs), respectively. In the original NCA scheme, the topology information *Z*_0 _is provided by the ChIP-on-chip data [9]. With the ChIP-on-chip data available in yeast, NCA has been successfully applied to yeast stress response and cell cycle experiments. Among the estimated TFAs with an oscillation pattern, 75% correspond to known cell-cycle regulators [7]. However, this NCA scheme is not readily applicable to many other biological studies due to the lack of topology information. In the next section, we will use motif information as a practical means to obtain the initial topology information for NCA.

### Motif analysis for initial topology information

A transcription factor (TF) is a protein that regulates its target gene's transcription by binding to a specific regulatory motif in the DNA of the promoter region(s). Thus, we can utilize regulatory motif information to establish the putative topologic relationship between a TF and a downstream target gene. Below we propose a motif analysis procedure to obtain the initial topology information for network reconstruction.

First, the upstream regions of the genes can be extracted from the database PromoSer [10]. Second, Match™ [11] (or its improved version, P-Match [12]) can be used to search the transcription factor binding sites (TFBSs) in each upstream region; this approach generates the scores of both "core similarity" and "matrix similarity" for each matched motif. Third, Match™ searches the TFBS for its position-weighted matrices (PWMs) that can be extracted from the TRANSFAC 11.1 Professional Database [13]. Fourth, according to the PWMs, a motif score can be calculated for each TF-gene pair where the score is the maximum of the average scores of core similarity and matrix similarity. These motif scores provide the initial topology information for further mNCA analysis as is detailed in the next section.

Note that each motif is a relative short sequence pattern, thus the topology from motif information is merely a rough estimation and will usually include many false positives/negatives. While the topology information is often unreliable for any specific TF-gene pair, we can still infer some key transcription factor activities from gene expression and DNA sequence information using the stability analysis procedure developed in the next section.

### Stability analysis for motif-directed NCA

Stability analysis was originally proposed to perform model selection for unsupervised learning, where the number of clusters can be correctly estimated [14]. Previously, we have developed a stability analysis procedure to estimate the dimension for linear decomposition problems [15]. The basic idea of stability analysis is that if a small perturbation is introduced equally in different model order, the best consistency will only occur when the model fits correctly the underlying structure of the data.

Here we develop a stability analysis procedure to assess the estimation results of mNCA. Since true functional data on TFAs are usually unavailable, we must establish whether an estimated TFA is a reliable estimate or if this prediction has arisen by error or by chance. When the topology information, either from motif analysis or ChIP-on-chip data, contains many false positives/negatives, we must also determine which TFAs are the reliable estimates of underlying transcription factor activities, or whether these are simply random outcomes.

If we intentionally perturb the network topology, each of the estimated TFAs will change. A falsely or poorly estimated TFA tends to be altered easily by small perturbations and will appear to be unstable. On the contrary, a good TFA estimation, reflecting the consistency between microarray expression data and topology knowledge, will tend to keep its activity pattern throughout multiple perturbations. Therefore, random perturbations should be performed multiple times to test the stability of each predicted TFA.

We propose two stability analysis strategies for our motif-directed NCA scheme. Both strategies estimate whether the predicted TFAs are stable or not when we intentionally alter the motif connection information. The perturbation methods are described as follows:

1. A TF-gene connection is deleted if the motif score is below a predetermined cut-off threshold. By setting different cut-off thresholds, we can change the number of connections and so perturb the network topology. The higher the motif score cut-off is set, the fewer the number of predicted connections.

2. Regardless of the motif score, for each transcription factor its TF-gene connections are randomly altered by either deleting the existing connections or by inserting new connections with some small percentage (e.g., 10%).

For *K *independent connection perturbations and repeated runs, we will obtain *K *different estimates of the same TFA. Pair-wise absolute correlation is calculated between different runs, and the stability measurement is defined as follows:

stability measurements of *i*^th ^TFA = {|*CorrCoef*(*TFA*_*i*_(*j*), *TFA*_*i*_(*k*))|_*j*≠*k*_},

where *j *and *k *correspond to different perturbations, respectively. *CorrCoef*() is the Pearson correlation coefficient function. When stability measurements of a specific TFA are obtained, we can use several statistics including mean and variance estimates to describe a predicted TFA's robustness with respect to perturbation. In this paper, we use *boxplot *to visualize the stability measurement, simultaneously depicting its minimum, 25% percentile, median, 75% percentile, and maximum.

## Results and discussion

The proposed mNCA approach has been applied to analysing a time course microarray data set from an expression profiling study of muscle regeneration at Children's National Medical Center (CNMC) [16]. Muscle differentiation model has been widely used as a model system to study embryonic development and post-natal regeneration of muscle tissues. Although both *in vitro *and *in vivo *biological experiments of muscle differentiation were conducted and reported, to our knowledge, no computational approaches have yet been proposed to model muscle regeneration process. Below we report the experimental results from our data analysis and show that the mNCA approach can reveal important regulatory mechanisms in muscle regeneration.

### Data set description

Staged skeletal muscle degeneration/regeneration was induced by injection of cardiotoxin (CTX) as previously described [16]. Two mice were injected in gastrocnemius muscles of both sides, and then sacrificed at each of the following time points: 0, 12 h(ours), 1 d(ay), 2 d, 3 d, 3.5 d, 4 d, 4.5 d, 5 d, 5.5 d, 6 d, 6.5 d, 7 d, 7.5 d, 8 d, 8.5 d, 9 d, 9.5 d, 10 d, 11 d, 12 d, 13 d, 14 d, 16 d, 20 d, 30 d, and 40 d. The time course microarray data set was acquired with Affymetrix's Murine Genome U74v2 Set from an expression profiling study at CNMC. We used Affymetrix's MAS 5.0 probe set interpretation algorithm to process the original intensity data for gene expression measurements. After the processing, we obtained the expression measurements of 7570 probesets in each sample.

### Motif analysis for topology information

From the TRANSFAC 11.1 Professional Database, 24 mouse muscle related transcription factors were selected for motif analysis (Table 1). According to their position weighted matrices (PWMs), possible connection topology was calculated. As described in the previous section, each possible connection has a motif score obtained from the TRANSFAC database. After motif information filtering, a total of 5198 genes were kept for further analysis using mNCA.

**Table 1:** Mouse muscle related transcription factors obtained from the TRANSFAC 11.1 Professional database.

Index	Regulatory Site	TRANSFAC Matrix ID	Brief Description
1	YY1	M01035, M00059, M00069, M00793	Ying Yang 1; common factor 1; delta-factor; F-ACT1; myc-CF1; NF-E1
2	Tal-1 alpha:E47	M00066	Tal-1beta:E47 heterodimer. Random 35-mers bound by in vitro co-translated Tal-1alpha and E47 after 6 CASTing cycles
3	NF-Y	M00775, M00185, M00287, M00209	Nuclear Factor Y; CCAAT-binding factor; CP1
4	Alpha-CP1	M00687	alpha-CP1; CBF; CCAAT-binding factor; CP1
5	Sp1	M00008, M00196, M00932, M00931, M00933	Stimulating Protein 1; trans-acting transcription factor 1
6	Hand1:E47	M00222	Hand1:E47 heterodimer. Hand1 is thought to bind to the left half (positions 1–8), E47 to the right half (position 9–16)
7	MEF-2	M00405 M00006, M00232, M00406, M00941, M00233, M00231	Myogenic enhancer factor 2
8	USF	M00796, M00187, M00217, M00121, M00122	Upstream stimulating factor
9	USF2	M00726	Upstream stimulatory factor 2; Fos-interacting protein
10	Tal-1beta:E47	M00065	Tal-1alpha:E47 heterodimer. Random 35-mers bound by in vitro co-translated Tal-1beta and E47 after 6 CASTing cycles
11	Ebox	M01034	Ebox binding protein
12	Myogenin	M00712	Myf-4 (human); MyoG; myogenin
13	E2A	M00804, M00973	E2-alpha; immunoglobulin enhancer binding factors E12/E47
14	NKX25	M01043	Csx; NK2 transcription factor related, locus 5 (Drosophila)
15	Nkx2-5	M00240, M00241	Homeo domain factor Nkx-2.5/Csx, tinman homolog
16	TATA	M00252, M00216	TATA-binding protein; TATA-box-binding protein; TBP; TFIID; TFIIDtau
17	TBX5	M01019, M01020, M01044	T-box protein 5
18	MyoD	M00001, M00184, M00929	Myoblast determining factor
19	SRF	M00152, M00810, M01007, M00186, M00215, M00922	Serum Responsive Factor
20	TBP	M00471, M00980	TATA-binding protein
21	GATA-4	M00632	GATA-binding factor 4
22	GATA	M00789	GATA-binding factor
23	E47	M00071, M00002	E2A; immunoglobulin enhancer-binding factor E12/E47
24	E12	M00693	E2A; immunoglobulin enhancer-binding factor E12/E47

### Motif-directed NCA and stability analysis

From Equation (3), we know that log-ratio operation should be performed on the data set to ensure that the linear model holds. We chose the last (27^th^) time point sample as the reference for calculating the ratios, because it is at the 40^th ^day, the late stage of the muscle regeneration, hence being regarded as a normal muscle reference.

**Figure 2:**
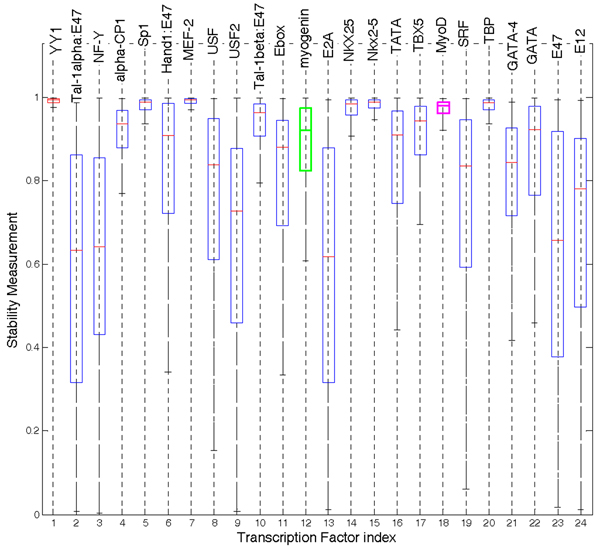
Stability measurements using the first perturbation procedure. The boxes with red, green purple colour are the stability measurements of YY1, myogenin and MyoD, respectively.

As described in Methods, two different perturbation procedures for mNCA were used to study the stability of estimated TFAs. In both procedures, the number of connection perturbations for mNCA was set to 20. A procedure to select a proper number of perturbations will be given later. In the first procedure, the threshold of motif score was set from low to high, making the connection number vary gradually from 12,000 to 18,000; this approach results in more than 30% of the connections being altered. Stability measurements were calculated and the boxplot generated (Fig. 2). It can be seen from Fig. 2 that some of estimated TFAs are stable during perturbation. Among them, the TFAs of YY1, myogenin and MyoD are quite stable as marked with different colours (Fig. 2).

**Figure 3:**
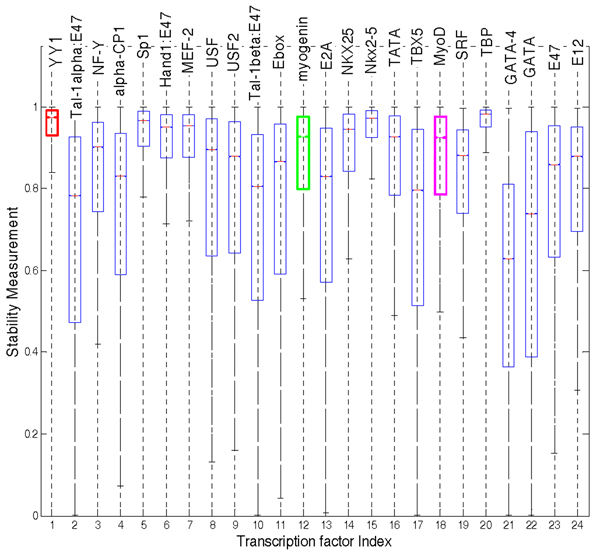
Stability measurements using the second perturbation procedure. The boxes with red, green, purple colour are the stability measurements of YY1, myogenin and MyoD, respectively.

In the second procedure, for each transcription factor, 10% of the connections were altered randomly, regardless of the motif score, by either deleting existing connections or inserting new connections to test the stability of TFAs. The stability measurement was calculated; the resulting boxplot is shown in Fig. 3. Again, the estimated TFAs of YY1, myogenin, and MyoD are seen to be stable as highlighted with different colours in Fig. 3.

To properly select the number of perturbations, we also investigated the stability measurements of TFAs with different numbers of perturbations. This should provide some justification on selecting a proper number for perturbations (denoted as NP). As summarized in Table 2, we report the experimental results on YY1, mygenin and MyoD with 25% quantile, median and 75% quantile for NP = 10, 15, 20, 30 and 50. As we can see, the YY1, myogenin and MyoD are very stable and consistent throughout the perturbations (see additional file 1: Stability analysis using mNCA with different number of perturbations). Therefore, we can justify that the selection of NP = 20 is a reasonable choice for this study.

**Table 2:** A summary of the stability analysis using mNCA with different number of perturbations for YY1, myogenin and MyoD.

Stability Measurement of transcription factor's activity/No. of perturbations		10	15	20	30	50
YY1	75% quantile	0.9981	0.9978	0.9977	0.9979	0.9977
	**median**	**0.9963**	**0.9960**	**0.9948**	**0.9959**	**0.9947**
	25% quantile	0.9928	0.9913	0.9886	0.9905	0.9870

myogenin	75% quantile	0.9776	0.9623	0.9745	0.9633	0.9662
	**median**	**0.9359**	**0.9092**	**0.9223**	**0.9107**	**0.9022**
	25% quantile	0.8680	0.8110	0.8250	0.8078	0.7959

MyoD	75% quantile	0.9908	0.9898	0.9894	0.9904	0.9894
	**median**	**0.9793**	**0.9816**	**0.9808**	**0.9816**	**0.9780**
	25% quantile	0.9632	0.9634	0.9621	0.9620	0.9542

### Discussion

**Figure 4:**
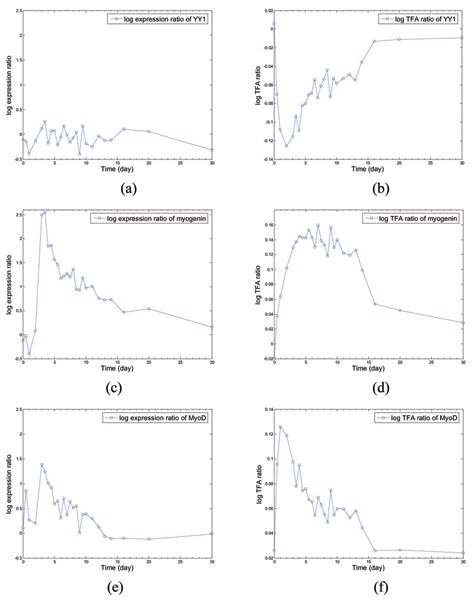
Gene expression patterns of (a) YY1, (c) myogenin, and (e) MyoD, respectively; estimated TFAs of (b) YY1, (d) myogenin, and (f) MyoD, respectively. Note: x-axis – time points; y-axis – log expression ratio (a, c and e) or log TFA ratio (b, d, e).

Because motif information is too general to fit to specific muscle regeneration data, stability analysis was used to find those transcription factors with stable estimated activities throughout perturbation. Although there are more than ten stable TFAs from the analysis, we focus here on three: MyoD, myogenin, and YY1. From the literature, these three TFs are key regulators of muscle differentiation [16-18]. In Fig. 4, we show the expression profiles and corresponding TFAs of these three TFs. It can be seen from Fig. 4 that these predicted TFAs are biologically relevant to muscle regeneration because the TFAs exhibit sudden increases in their log expression ratios after muscle injury and these values gradually decrease in the later stages of muscle regeneration when the tissue has almost completed regeneration.

**Figure 5:**
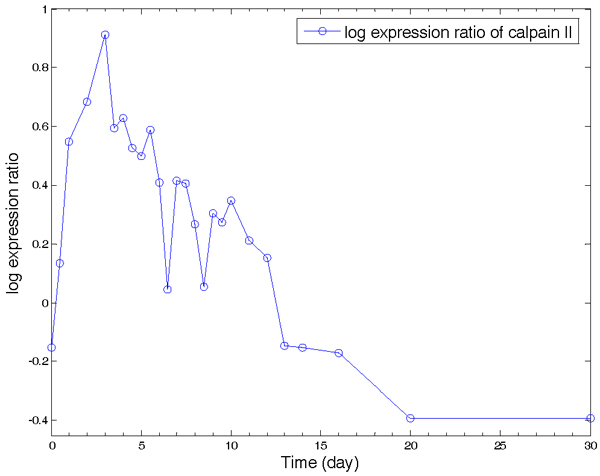
Gene expression pattern of calpain II. Note: x-axis – time points; y-axis – log expression ratio.

For YY1, a large difference between its measured gene expression level and inferred TFA is evident in Fig. 4(a) and Fig. 4(b). The YY1 gene expression log-ratio is relative low when compared with other TFs, and its trend has no obvious relationship with muscle regeneration. However, the inferred TFA shows a close relationship with the regeneration process. This is supported by a biological study [18] that reported an inconsistency between YY1 protein and mRNA expression levels and showed an important role for YY1 in mouse muscle differentiation. Specifically, YY1 acts as a transcription repressor, down-regulating muscle gene expression in undifferentiated muscle cells [19]. During muscle differentiation, YY1's activity is decreased, which leads to the induction of muscle gene expression. The reduction in YY1 activity occurs at the protein rather than mRNA level. YY1 protein is degraded by a protease, calpain II (m-calpain), in differentiating muscle cells [16]. Thus, our inferred YY1 TFA from the muscle regeneration data set is well supported by the biological observation of an induction of calpain II and relatively less change of YY1 mRNA expression in muscle regeneration. It can also be observed that calpain II's mRNA expression levels (Fig. 5) have a very similar pattern with our estimated YY1 TFA (Fig. 4(b)), with a correlation coefficient of r > 0.9.

Intuitively, a regulator's TFA should have better prediction capability to describe its target genes' pattern than using its own mRNA expression level. From ChIP-on-chip experiments of myogenin [17], more than one hundred genes were identified as its regulation target candidates. Testing their relationship by a simple Pearson correlation calculation, there are 14 probeset IDs with an absolute correlation coefficient (ACC) > 0.8 with myogenin's TFAs; only 6 probeset IDs correlated (ACC > 0.8) with the myogenin's measured expression level. We note that myogenin's mRNA expression level and its TFA estimate have a similar pattern; the correlation results show that the estimated TFA is better able to predict downstream targets than its measured gene expression level. These observations indicate that our mNCA approach has significant potential to find better regulation targets for pathway discovery.

## Conclusion

In this paper, we propose a new approach, namely motif-direct NCA (mNCA), to infer underlying regulatory activities by integrating motif information and gene expression data. Motif information is used to derive initial network topology information for mNCA. Since many false positives/negatives could exist in motif information, we have further developed a stability analysis procedure for mNCA to extract stable TFAs. The scheme was applied to a time-course microarray data set from a muscle regeneration profiling study. The experimental results show that our new approach can reveal both key regulators and their target genes, and also discover novel regulatory mechanisms potentially involved in muscle regeneration. By further incorporating biological knowledge, we hope to extend this approach to analyzing muscle dystrophy data for novel pathway discovery and biomarker identification [8].

## Competing interests

The authors declare that they have no competing interests.

## Authors' contributions

CW and JX formulated the problem and developed the theoretical framework of the algorithm. CW and LC carried out the development and implementation of the algorithm. PZ and EH directed the application of the algorithm to muscle regeneration data. YW and RC provided technical and biological support to the project. All authors participated in the writing of the manuscript, and have read and approved the manuscript.

## Supplementary Material

Additional file 1 Stability analysis using mNCA with different number of perturbations (denoted as NP). Stability measurements with (a) NP = 10, (b) NP = 15, (c) NP = 20 and (d) NP = 30. The boxes with red, green, purple colour are the stability measurements of YY1, myogenin and MyoD, respectively. Click here for file
