# Supplementary material for: Motif-directed network component analysis for regulatory network inference
Source: BMC Bioinformatics. 2008 Feb 13;9(Suppl 1):S21. doi: 10.1186/1471-2105-9-S1-S21 (PMC2259422; doi:10.1186/1471-2105-9-S1-S21)
Supplement: Additional file 1 — Stability analysis using mNCA with different number of perturbations (denoted as NP). Stability measurements with (a) NP = 10, (b) NP = 15, (c) NP = 20 and (d) NP = 30. The boxes with red, green, purple colour are the stability measurements of YY1, myogenin and MyoD, respectively. [file 1471-2105-9-S1-S21-S1.pdf]

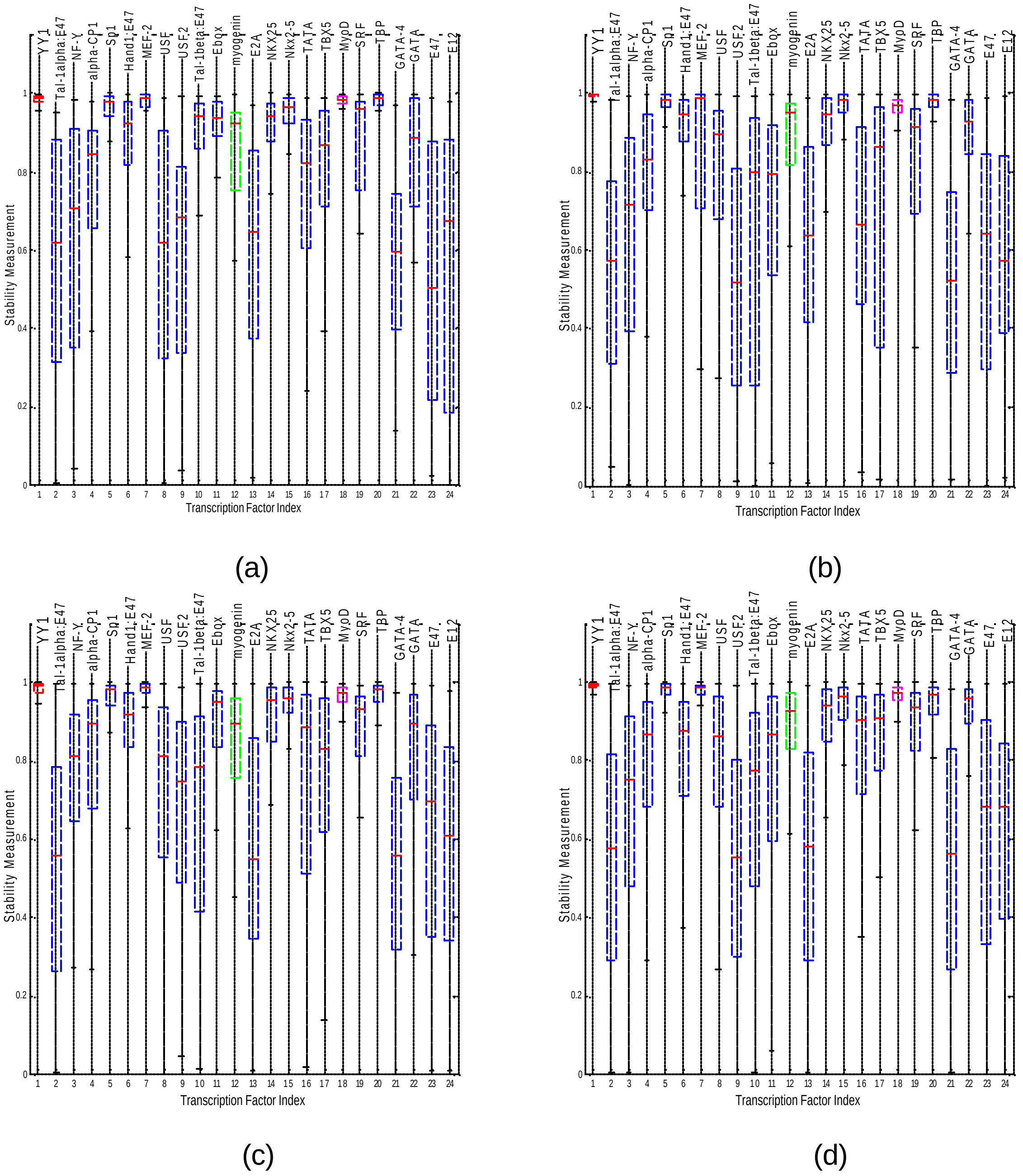

Additional file 1: Stability analysis using mNCA with different number of perturbations (denoted as NP). Stability measurements with (a) NP = 10, (b) NP = 15, (c) NP = 20 and (d) NP = 30. The boxes with red, green, purple colour are the stability measurements of YY1, myogenin and MyoD, respectively.
